# 922. Psychiatric Disorders in Burn Injury: A Systematic Review and Meta-analysis

**DOI:** 10.1093/jbcr/irag033.447

**Published:** 2026-04-06

**Authors:** Diana B Griffin, Marlynn P Lopez, Emily Duckworth, Angelica Bartler, Aneeq Chaudhry, Robert Galiano

**Affiliations:** Northwestern University, Chicago, IL; Northwestern University, Chicago, IL; University of South Carolina School of Medicine Greenville, Greenville, SC; Chicago Medical School - Rosalind Franklin University of Medicine and Science, Antioch, IL; Northwestern University, Chicago, IL; Northwestern University, Chicago, IL

## Abstract

**Introduction:**

Burn injuries are associated with profound and enduring functional and psychological consequences that can persist for years despite advances in clinical practice that improve survival. This study aims to assess the role of psychiatric diagnoses prior to burn, the development of psychiatric diagnoses after burn, as well as the relationship between patient and burn characteristics and the development of a new psychiatric diagnosis after a burn injury.

**Methods:**

This study has an independent Prospero registration. A literature search was conducted in September 2025, using the Embase, Ovid, PubMed, and Cochrane Library databases to identify observational studies involving adults who experienced burn injuries. Case reports, case series, reviews, editorials, letters to the editor, animal studies, and in vitro studies were excluded. Results were synthesized using random-effects models to generate pooled odds ratios (ORs) with 95% confidence intervals. Risk of bias assessments and sensitivity analysis were conducted.

**Results:**

A total of 2654 articles were identified; 159 studies were selected for the systematic review, and of these, 36 provided sufficient data for meta-analysis. The presence of any pre-existing psychiatric diagnosis is associated with increased likelihood of a burn injury (OR 6.46 CI 95% 1.54-27.16, I2 = 98.3%, τ^2^ = 1.5762, Q(2) = 120.06, *p*=.0000). After a burn injury, there are increased odds of mental health related hospital admissions (OR 2.98, 95% CI 2.04-4.33, I2 = 88.9%, τ^2^ = 0.0658, Q(1) = 8.98, *p*=.0027) and the development of a new psychiatric diagnosis (OR 1.75, 95% CI 0.99-3.10, I2 = 99.6%, τ^2^ = 0.4920, Q(5) = 1241.93, *p*=.0000). Burn patients are more likely to develop depression (OR 1.57, 95% CI 1.11-2.21, I2 = 96.9%, τ^2^ = 0.1818, Q(6) = 196.08, *p*=.0000), anxiety (OR 2.34 CI 95% 1.11-4.95), substance use disorder (OR 4.76 CI 95% 1.33-16.97), and PTSD (OR 4.49 CI 95% 2.43-8.28).There are no significant differences with regards to burn type or body site across those with and without a pre-existing psychiatric diagnosis and only weak associations with patient characteristics including marital and employment status.

**Conclusions:**

This review suggests there is insufficient data and weak evidence to identify individuals who will develop a psychiatric diagnosis. However, this review suggests that patients with pre-existing psychiatric diagnosis are more likely to develop a burn and that patients are at increased odds to develop a new psychiatric diagnosis after a burn injury.

**Applicability of Research to Practice:**

These findings support the need for universal mental health screening at admission and throughout recovery, with early consult-liaison psychiatry involvement and regular post-discharge follow up. Patients with known psychiatric illness should receive targeted injury-prevention counseling and regular mental health support.

**Funding for the study:**

N/A.

